# Demyelinating syndrome in systemic sclerosis and neuromyelitis optica

**DOI:** 10.1186/s12883-019-1472-6

**Published:** 2019-10-14

**Authors:** Khaled Deeb, Jessika Eby, Jose Labault-Santiago

**Affiliations:** 1 Miller School of Medicine, Internal Medicine Residency, University of Miami, 5301 S Congress Aven, Atlantis, FL 33426 USA; 2 Miller School of Medicine, Palm Beach Regional Campus, University of Miami, Atlantis, FL USA; 3 Department of Neurology, JFK Medical Center, Atlantis, FL USA

**Keywords:** Neuromyelitis optica, Systemic sclerosis, Demyelinating syndrome, Systemic lupus erythematous

## Abstract

**Background:**

This article reports a case diagnosis of a 44-year-old female who presented with intractable hiccups and vomit complicated with an acute onset of paraplegia. Transverse myelitis was evident on MRI and serological studies were consistent with Neuromyelitis Optica (NMO) based on NMO-IgG sero-positivity. Further studies revealed positive ANA, anti-RNA polymerase III autoantibodies, and Scl-70, leading to a concurrent diagnosis of systemic sclerosis (SSc). The coexistence of these two disease processes and their underlying clinical manifestations and therapeutic interventions are seldom reported in literature and are worth reporting.

**Case presentation:**

The patient was treated with high dose steroids, and subsequently developed malignant hypertension and acute renal failure, later identified on biopsy as steroids-induced scleroderma renal crisis. Although Neuromyelitis Optica spectrum disorder (NMOSD) has often been associated with various collagen and autoimmune diseases, the coexistence of NMOSD and SSc presented a challenge where the patient underwent aggressive physical therapy and necessitated an intervention with Rituximab to achieve an appropriate clinical response.

We have received a written consent forms from the participant in our study, and we have them on file in case they are requested. We have also received the patient’s written consent for the data and images presented in this article.

**Conclusion:**

This article expands on NMOSD associated autoimmune diseases. Systemic Sclerosis is an insidious disease that is often diagnosed late as not all patients often report skin manifestation. The finding suggests that patients presenting with acute neurological manifestations get tested for NMO-IgG/AQP-4 antibodies and other immunological studies based on clinical findings.

## Background

Neuromyelitis optica (formerly known as Devic’s disease) is an inflammatory disorder of the CNS affecting predominantly the spinal cord, brainstem, and optic nerves. Typical presentation includes acute optic neuritis (leading to vision loss), transverse myelitis (leading to limb weakness, sensory loss), or brainstem syndromes (intractable hiccups, nausea, vomiting). Intractable hiccups and nausea are rare and unique symptoms in NMO, and they are believed to be due to involvement of area postrema and nucleus tractus solitarius in the dorsomedial medulla and ventrolateral respiratory center [1, 2].

Neuromyelitis optica spectrum disorders are rare, constituting less than 1% of demyelinating disease with a significantly higher number of female than male patients. The incidence and prevalence are based on population-based studies conducted in Europe, South East and Southern Asia, the Caribbean, and Cuba ranged from 0.05 to 0.4 and 0.52 to 4.4 per 100,000, respectively [3]. It was previously believed to be a variant of multiple sclerosis (MS), as it shares both clinical and radiologic features [2, 4]. However, it is now considered a separate entity after the discovery of disease-specific NMO-IgG antibody that selectively binds aquaporin-4 (AQP4). It is thus crucial to differentiate between MS and NMO because Immunosuppressive drugs (eg, azathioprine and corticosteroids) are regarded as the best treatment for NMO, whereas immunomodulatory treatments (eg, interferon beta and glatiramer acetate) are presently recommended for early treatment of MS. Our case of NMO diagnosis is based on the revised consensus criteria in 2015 [5], with the presence of six clinical features, in conjunction with antibody seropositivity and additional MRI findings, as shown in Table 1.

**Table 1:** Titers and lab values

Test name	Titers	Reference ranges
	Neuromyelitis Optica... 7.7 A	Negative 0.0–3.0;Indeterminate 3.1–5.0;Positive > 5.1
	ANA Positive	
	ANTI-DNA < 1.0	Negative: <=4.9 IU/mL;Indeterminate: 5–9.9 IU/mL;Positive: > = 10 IU/mL
Smooth Muscles	SM AB < 0.2	Negative: < 1.0;Positive: > = 1.0
Neutrophil Cytoplasmic Ab	C-ANCA < 0.2, P-ANCA < 0.2	Negative: < 1.0;Positive: > = 1.0
Scleroderma 70 Ab	SCLERODERM... 4.5 H	Negative: < 1.0;Positive: > = 1.0
	JO-1 ANTIB... < 0.2	Negative: < 1.0;Positive: > = 1.0
	CENTROMERE AB... < 0.2	Negative: < 1.0;Positive: > = 1.0
Sjorgen	ANTI-SSA... < 0.2, ANTI-SSB... < 0.2	Negative: < 1.0;Positive: > = 1.0
	RNP AB < 0.2	Negative: < 1.0;Positive: > = 1.0
	CHROMATIN ... < 0.2	Negative: < 1.0;Positive: > = 1.0
	RA Negative	
	HBsAg Scre... NEGATIVE	
	HEP B SURF... NEGATIVE	
	RNA Polymerase III IgG Abs 40 Units/mL	Negative: < 20;Weak Positive: 20–39;Moderate Positive: > 40–80;Strong Positive: > 80^a^
	C3 131	
	C4 40	
HIV By By RT-PCR	HIV RNA... Target Not Detected	
Toxoplasma Igg	TOXOPLASMA... 27.0 H,	
	TOXOPLASMA... 0.24	< 0.9 Negative;0.9–0.99 Equivocal;> 1.00 Positive
	MYELIN BASIC PROTEIN... 6.3 A	
	EBV EARLY ... < 0.20, EBV NUCLEA... > 8.00 H,	
	EBV VCA IG... > 8.0 H, EBV VCA IG... < 0.2	< OR = 0.8 AI: NEGATIVE;0.9–1.0 AI: EQUIVOCAL;> OR = 1.1 AI: POSITIVE
	VARICELLA IgG … .3.3	< OR = 0.8 AI: NEGATIVE;0.9–1.0 AI: EQUIVOCAL;> OR = 1.1 AI: POSITIVE
	HTLV I/II ... TNP	
Epst Barr Virus DNA	Epst Barr ... Negative	
Varicella Zoster Ab Pcr (Csf)	VARICELLA ... Negative	
Crypto.Ag,Csf	CRYPTO.AG,... Negative	
Herpes Virus Dna By Pcr	HSV1 DNA P... Negative, HSV2 DNA P... Negative	
Lyme Disease Ab (Csf)	B.burgd Ig... < 0.08, B.burgd Ig... < 0.06	Negative: < or = 0.09;Positive: > 0.09B.
	Enterovirus... Negative	
	CSF VDRL Nonreactive	
	IGG 1500	
	Coccidioides Abs, CSF (CF) < 1:2 < 1:2	
	Coccidioides Ab, IgG 0.1 IV < =0.9	
	Toxo IgG C... < 3.0	
Oligoclonal	OLIGOCLONAL ab negative	
	GLUCOSE, C... 99 H; PROT.,CSF 55.6 H	
Immunofix/Pep Csf Profile	PROTEIN (C... 36.9, PREALBUMIN 2.9,	
	ALBUMIN 54.5 A, ALPHA-1-GL... 4.1, ALPHA-2-GL... 6.4,	
	BETA GLOBU... 17.8, GAMMA GLOB... 14.4 A	
Lymph/Leuk Body Fluid	plasma cells are not identified	T cells with an increased CD4/CD8 ratio are detected. ^b^
	ANGIOTENSIN ENZYM < 15	
	Renin Activity... 13.774 A	
	D-DIMER 476 H	
	CORTISOL 34.66	
	TSH 0.910	
	ESR 86 H	
	CRP-HIGH S... 1.910 H	

^a^RNA Polymerase III is strongly associated with diffuse cutaneous scleroderma and with an increases risk of acute renal crisis. A positive result supports a possible diagnosis of systemic sclerosis while a negative result does not rule out the possibility of systemic sclerosis, as the sensitivity for detection of RNA Polymerase III is between 11 and 23%

^b^Flow cytometric analysis identifies a population of lymphocytes (91.1% of all analyzed events) that consists of a mixture of minute B-cell population (2.7% of cells in this gate), T cells with an increased CD4/CD8 ratio of 7.8 and mild down-regulation of CD7 expression (91.6% of cells in this gate), and NK-cells (2.0% of cells in this gate). An increased CD4/CD8 ratio is a non-specific finding that may be seen in reactive / inflammatory processes and autoimmune disease; less likely, it may represent an atypical population. The B-cells show minimal lambda excess, the significance of which is uncertain

Neuromyelitis optica is known to coexist with other connective tissue and autoimmune disorders (e.g., SLE, Sjögren); however, it has no prior conjunction with Systemic Sclerosis (SSc). One may confirm the positivity of NMO by having NMO-IgG/AQP-4 antibodies. Serum detection of specific autoantibodies, such as anti-dsDNA, is the hallmark of SLE, and anti-topoisomerase antibodies (SCL-70) are the most specific test for SSc, as they may help in the differential diagnosis of the first demyelinating episode. Demyelinating syndromes could be life threatening and may lead to disability and even death. The estimated prevalence of SLE is 1.3% and incidence rate of 1.5 case per 1000 patients [6]. It was found that IFNs play an important role in the pathogenesis of connective tissue diseases where it was noted that serum IFN-α/β activity is elevated in SLE as well as NMO, which is no surprise due to the similarities in pathophysiology [7]. It was also found that the higher the IFN activity, the more severe is the SLE disease [8]. Furthermore, Patients with gray matter dysfunction had higher levels of SLE activity, whereas patients with white matter dysfunction were more likely to meet criteria for NMO [9]. On the other hand, SSc is another form of autoimmune rheumatic disease described by having excess collagen deposition in the skin and viscera, along with vascular abnormalities including vasospasm and microvascular occlusion [10]. While uncommon, neurological complications of SSc has been reported with manifestations of peripheral neuropathy with sensitive-motor forms [11–14].

## Case presentation

This case presents a 44-year-old Hispanic female, with no known past medical history, complaining of an acute onset of intractable hiccups, vomiting, and dysphagia. Physical examination was notable for a thin female appearing much younger than stated age. Cardiac and pulmonary examinations were unremarkable. Skin thickening of the fingers extending proximal to the metacarpophalangeal joints was noted bilaterally. Review of systems was positive for Reynaud’s phenomenon, however negative for finger ulceration, nail pitting, dry eyes or mucus membranes, or skin rash. Vital signs on admission were stable. Laboratory studies were unremarkable except for hyponatremia and hypokalemia, which were replaced appropriately. Patient received supportive IV fluids and anti-emetics. The following day, the patient developed new onset of blurry vision, motor weakness and paresthesias of the bilateral lower limbs. Lower extremities were notable for hypertonia and paroxysmal tonic spasms. Motor strength of lower extremities was 0/5 with intact bilateral upper extremity strength. Upper extremity reflexes were 2+, and knee and ankle reflexes were 4+ bilaterally. Babinski’s sign was present bilaterally. Loss of pinprick and vibratory sensation up to bilateral anterior superior iliac spine was noted. Visual acuity diminished bilaterally with no visual field defects, and extra ocular movement was intact. Other cranial nerves were intact. Brain MRI with and without gadolinium enhancement showed multiple scattered hyper-intense T2 flair foci in the bilateral periventricular white matter. MRI spine revealed multiple enhancing lesions in the spinal cord at the levels of C2–C6, consistent with transverse myelitis.

Extensive laboratory evaluation, which included thyroid function, vitamin B12 level, and folic acid was normal. Serologies and PCRs for common neurotropic viruses (HSV1, HSV2, CMV, EBV, HBV, HCV, VZV, HTLV 1/2, Toxoplasma, and HIV) were negative in blood and in cerebrospinal fluid analysis (CSF). Specifically, CSF analysis revealed lymphocytic pleocytosis but no oligoclonal bands. Immunological tests for anti-dsDNA, anti-cardiolipin, anti-β2GPI, ANCA, and cryoglobulins were negative. However, ANA, anti-RNA polymerase III autoantibodies, Scl-70, and NMO-IgG were all positive. Repeat NMO-IgG was positive. Effectively, preliminary diagnosis of NMO was made based on seropositivity and clinical features consistent with transverse myelitis and area postrema syndrome (see Table 1). Our case of NMO diagnosis is based on the revised consensus criteria in 2015, shown in Table 2.

**Table 2:** Neuromyelitis optica spectrum disorders diagnostic criteria for adult patients

Diagnostic criteria for NMOSD with AQP4IgG	
1. At least one core clinical characteristic	
2. Positive test for AQP4IgG using best available detection method (cell-based assay strongly recommended)	
3. Exclusion of alternative diagnoses	
Diagnostic criteria for NMOSD without AQP4IgG or NMOSD with unknown AQP4IgG status	
1. At least two core clinical characteristics occurring as a result of one or more clinical attacks and meeting all of the following requirements:	
a. At least one core clinical characteristic must be optic neuritis, acute myelitis with LETM, or area postrema syndrome	
b. Dissemination in space (two or more different core clinical characteristics)	
c. Fulfillment of additional MRI requirements, as applicable	
2. Negative tests for AQP4IgG using best available detection method, or testing unavailable	
3. Exclusion of alternative diagnoses	
Core clinical characteristics	
1. Optic neuritis	
2. Acute myelitis	
3. Area postrema syndrome: Episode of otherwise unexplained hiccups or nausea and vomiting	
4. Acute brainstem syndrome	
5. Symptomatic narcolepsy or acute diencephalic clinical syndrome with NMOSD typical diencephalic MRI lesions	
6. Symptomatic cerebral syndrome with NMOSD typical brain lesions	
Additional MRI requirements for NMOSD without AQP4IgG and NMOSD with unknown AQP4IgG status	
1. Acute optic neuritis: Requires brain MRI showing (a) normal findings or only nonspecific white matter lesions, or (b) optic nerve MRI with T2 hyperintense lesion or T1weighted gadolinium enhancing lesion extending over more than one half the optic nerve length or involving optic chiasm	
2. Acute myelitis: Requires associated intramedullary MRI lesion extending over ≥3 contiguous segments (LETM) or ≥ 3 contiguous segments of focal spinal cord atrophy in patients with history compatible with acute myelitis	

*AQP4* aquaporin4, *IgG* immunoglobulin G, *LETM* longitudinally extensive transverse myelitis lesions, *NMOSD* neuromyelitis optica spectrum disorders

The patient was started on high-dose intravenous methylprednisolone (1 g daily for five consecutive days) on day. Resolution of visual symptoms occurred within 2 days. Nonetheless, the patient showed marginal improvement in motor symptoms. On day 5 of hospitalization, patient developed abrupt onset of malignant hypertension, as well as acute onset of renal failure with non-nephrotic range proteinuria, requiring urgent hemodialysis. Renal biopsy showed obliteration of the vascular lumen, with concentric fibrinoid necrosis and “onion-skin” hypertrophy, most consistent with diagnosis of steroid induced scleroderma renal crisis. Steroids were discontinued and patient was started on an ACE inhibitor with resultant normalization of blood pressure and slow recovery of renal function. Patient was started on Azathioprine with improvement of motor symptoms; however, it was discontinued due to hepatotoxity. Effectively, it was changed to Rituximab 1000 mg × 2 doses 14 days apart, with gradual improvement in motor functions.

**Fig. 1:**
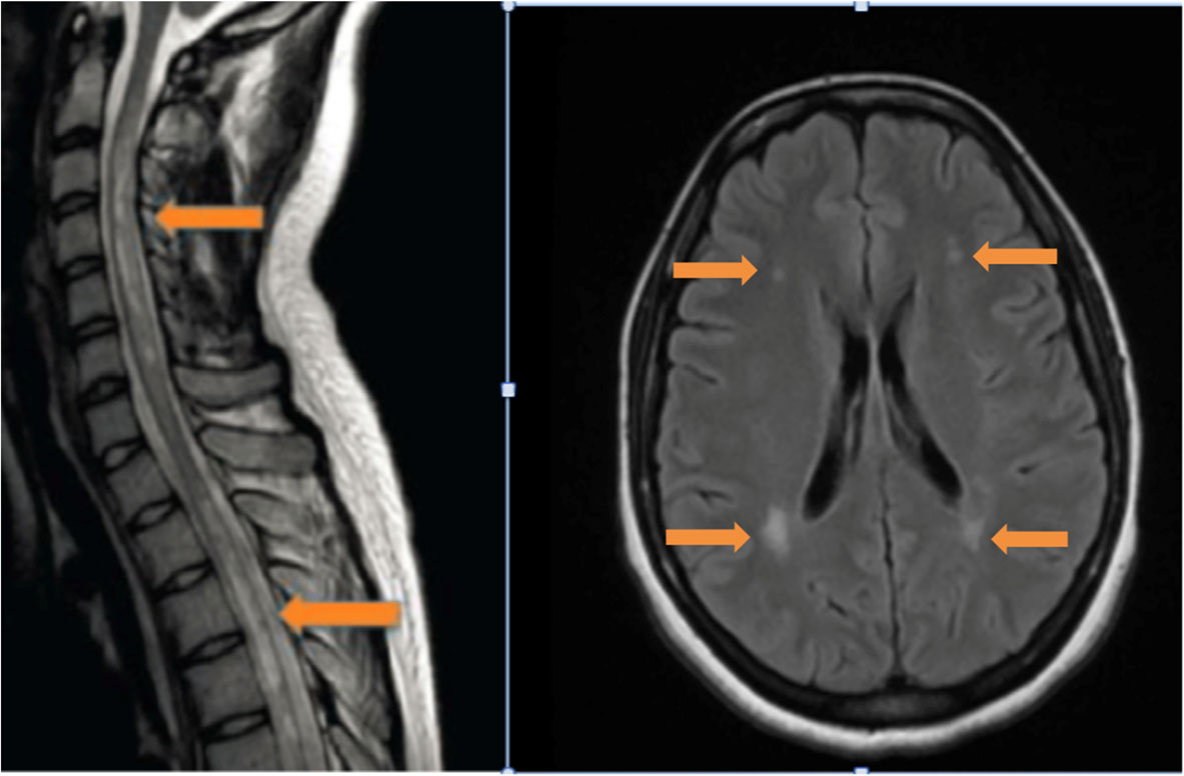
**a** T1-weighted sagittal MRI (left) of the cervical spinal and upper thoracic cord showing transverse myelitis related. Also noted extensive multiple areas of increased T2 signal abnormality within the thoracic spinal cord with patchy areas of post-contrast enhancement. **b** T2-weighted sequence MRI of the brain (right) show scattered bright foci of FLAIR demyelinating process in the bilateral deep periventricular and cerebral white matter and in the dorsum of medulla oblongata

In sum, our patient was diagnosed with both NMO and Systemic Sclerosis based on her history, clinical presentation, MRI findings as shown in Fig. 1, anti-Scl-70 antibodies, and the presence of serum NMO-IgG antibody. She had a subacute onset of lower extremity paraparesis, dysesthesia, and hyperreflexia, with gradual improvement in motor function over the course of a year. In the interim, she had multiple readmissions for painful paroxysmal tonic spasms which responded to Gabapentin, Baclofen and Carbamazepine.

## Discussion and conclusions

Neuromyelitis optica (NMO) and neuromyelitis optica spectrum disorders (NMOSD) are rare demyelinating disorders of the central nervous system that predominantly affects the spinal cord, brainstem and optic nerves [4]. While the cause of NMO is unknown, it has been hypothesized that an autoimmune-mediated inflammatory cascade leads to demyelination and axonal injury. Characteristics findings include optic neuritis, and episodic transverse myelitis. Association with other autoimmune disorders has been widely recognized, including systemic lupus erythematosus (SLE), sjögren syndrome (SS), rheumatoid arthritis (RA), and organ-specific autoimmune diseases (e.g., thyroid diseases, myasthenia gravis, ulcerative colitis) [15]. However, SSc-associated NMO is rarely reported in the literature. Systemic sclerosis is a systemic autoimmune disease characterized by excess collagen deposition in the skin and internal organs. Given that the etiology of NMOSD is not clearly understood, it is uncertain if SSc is an etiological factor in NMO.

This case highlights the need for proper diagnosis and treatment of coexisting autoimmune conditions, as well as the recognition of possible life-threatening side effects of conventional first line steroid therapy. Proper recognition also ensures prompt initiation of immunotherapy for motor and sensory recovery, as well as the need for comprehensive care of these patients in rehabilitation centers.

Although there have been several reports of NMO and SLE overlap syndrome responding to glucocorticoid therapy, treatment with steroid therapy in SSc should be avoided given the increased risk of scleroderma renal crisis. Alternatively, Rituximab was found effective for the prevention of attacks in one NMO case, resistant to cyclosporine therapy [9]. Immunoablative dose cyclosporine has also been successful in SLE-associated NMO in patients unresponsive to high-dose oral and intravenous corticosteroids, intravenous immunoglobulin, mycophenolate mofetil, tacrolimus, and rituximab [16]. Another case reported late onset of NMOSD after a long-standing SSc, where CSF studies showed positivity of anti-Smith antibodies and anti-Slc-70 antibodies. Disease modifying treatment was given with an infusion of rituximab [3].

We report a case of NMO overlapping with SSc without other organ involvement (e.g., GI, pulmonary, cardiac) except for steroid induced scleroderma hypertensive renal crisis and skin manifestation. Our patient fulfilled the criteria for both NMO and SSc given NMO-IgG and anti Scl-70 positivity in conjunction with typical physical findings and clinical presentation. Per guidelines, our patient was started on first-line immunosuppressive therapies (prednisolone and rituximab) and plasmapheresis. Although initially responding to plasmapheresis and rituximab therapy with improvement in motor function, she continued to experience disease relapses, including paraplegia and severe spastic muscle crisis. Muscle spasticity was improved with initiation of tizanidine and carbamazepine. Our patient had vigilant monitoring with timely intervention to address organ-based complications, namely arterial hypertension and scleroderma renal crisis.

## Data Availability

The datasets generated during and/or analyzed during the current study are available from the corresponding author on reasonable request.

## Abbreviations

MS: Multiple sclerosis
NMO: Neuromyelitis optica
NMOSD: Optica spectrum disorder
RA: Rheumatoid arthritis
SLE: Systemic lupus erythematosus
SS: Sjögren syndrome
SSc: Systemic sclerosis
